# Enlightening the malaria parasite life cycle: bioluminescent *Plasmodium* in fundamental and applied research

**DOI:** 10.3389/fmicb.2015.00391

**Published:** 2015-05-11

**Authors:** Giulia Siciliano, Pietro Alano

**Affiliations:** Dipartimento di Malattie Infettive, Parassitarie ed Immunomediate, Istituto Superiore di SanitàRome, Italy

**Keywords:** malaria, *Plasmodium*, bioluminescence, reporter genes, *in vivo* imaging, cell-based screening assays

## Abstract

The unicellular protozoan parasites of the genus *Plasmodium* impose on human health worldwide the enormous burden of malaria. The possibility to genetically modify several species of malaria parasites represented a major advance in the possibility to elucidate their biology and is now turning laboratory lines of transgenic *Plasmodium* into precious weapons to fight malaria. Amongst the various genetically modified plasmodia, transgenic parasite lines expressing bioluminescent reporters have been essential to unveil mechanisms of parasite gene expression and to develop *in vivo* imaging approaches in mouse malaria models. Mainly the human malaria parasite *Plasmodium falciparum* and the rodent parasite *P. berghei* have been engineered to express bioluminescent reporters in almost all the developmental stages of the parasite along its complex life cycle between the insect and the vertebrate hosts. *Plasmodium* lines expressing conventional and improved luciferase reporters are now gaining a central role to develop cell based assays in the much needed search of new antimalarial drugs and to open innovative approaches for both fundamental and applied research in malaria.

## Introduction

Half of the world population is at risk of malaria (World Health Organization, 2013), the most common, and severe parasitic mosquito-borne disease (White et al., 2014). Five species of the protozoan genus *Plasmodium* infect humans, with *Plasmodium falciparum* and *P. vivax* causing over 200 million cases/year and *P. falciparum* inflicting virtually all the 6–700,000 annual deaths (2013) recorded mainly in children of Sub-Saharan Africa.

**FIGURE 1:**
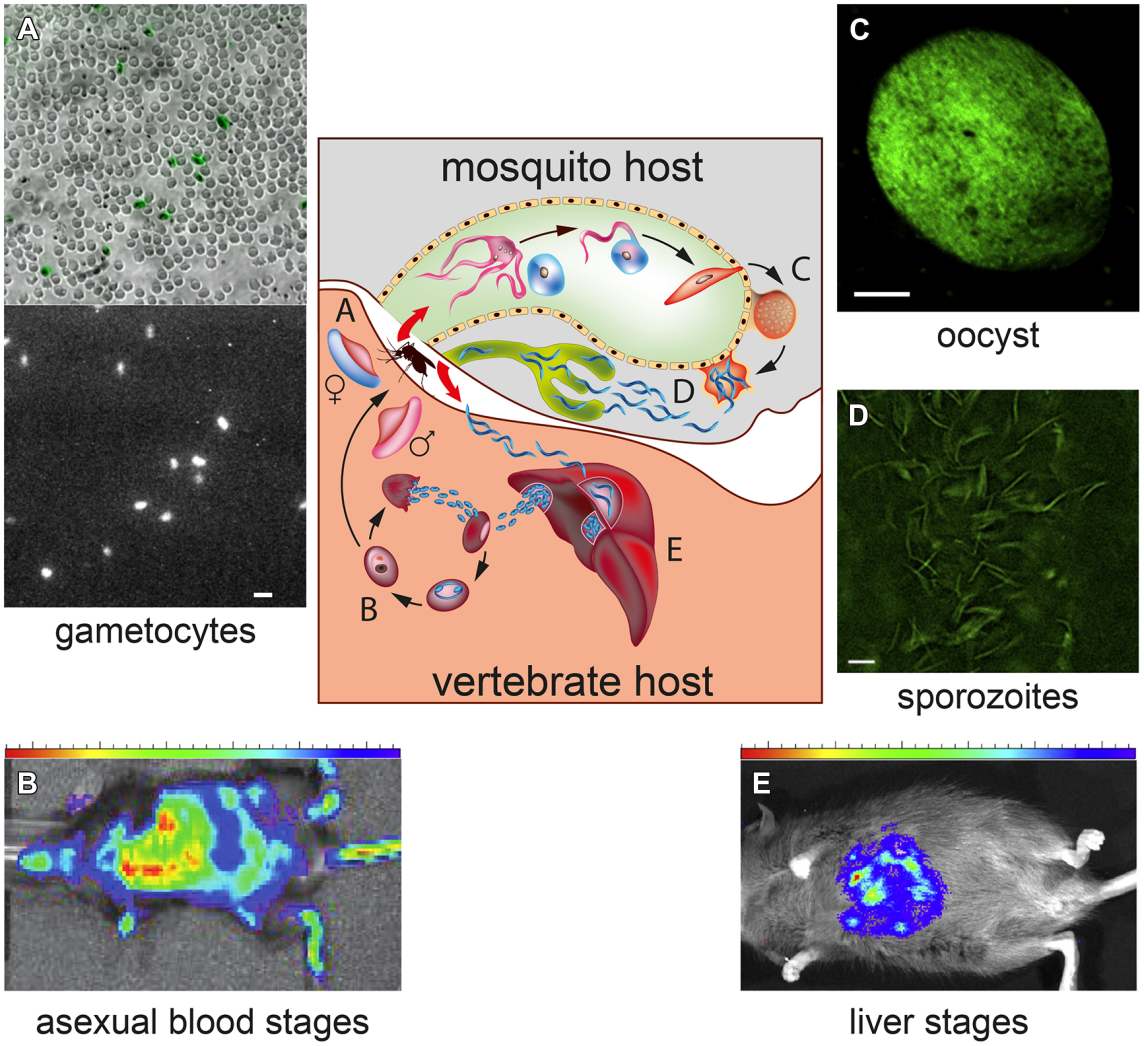
**Transgenic bioluminescent malaria parasites at different stages of their development in the vertebrate and mosquito hosts**. The central diagram represents the *Plasmodium* life cycle, showing the progression through the developmental stages of the parasites in the mosquito vector and in the vertebrate host. **(A)** Bioluminescence imaging (BLI) of individual *Plasmodium falciparum* gametocytes expressing a click beetle luciferase under a sexual stage-specific promoter. The bright field image shows immobilized gametocytes, highlighted in green, amongst uninfected erythrocytes; the dark field shows the bioluminescence signal of the gametocytes incubated with D-luciferin. Magnification bar: 15 μm. (Adapted from Cevenini et al., 2014). **(B)** BLI of a mouse infected with asexual *P. berghei* parasites expressing a firefly luciferase-green fluorescent protein (GFP) fusion. Heatmap of the bioluminescent signal identifies the sites of accumulation of the parasites (Reproduced with permission from Claser et al., 2011). **(C)** Fluorescence of a firefly luciferase-GFP fusion protein expressed in *P. falciparum* sporozoites contained in a oocyst and **(D)** obtained from the dissection of infected mosquito salivary glands. Magnification bar: 5 μm. **(E)** *In vivo* bioluminescent signal obtained by transgenic *P. falciparum* liver stage parasites developing in the chimeric liver of a humanized mouse **(C–E)** are reproduced with permission from Vaughan et al. (2012).

The malaria parasite exhibits a complex life cycle involving an *Anopheles* mosquito and a vertebrate host (**Figure 1**). When an infected female mosquito bites a human, the *Plasmodium* sporozoites travel to the liver and invade hepatocytes, where parasites replicate as hepatic schizonts until several thousand merozoites are produced and released in the bloodstream. In *P. vivax*, but not in *P. falciparum*, some liver parasites remain instead quiescent (hypnozoites), resuming replication, and infection after several weeks or months. Upon erythrocyte invasion in the bloodstream *Plasmodium* parasites undergo asexual replication forming mature schizonts whose rupture releases merozoites that invade new erythrocytes. Some blood stage parasites differentiate instead into male and female gametocytes that, when ingested in the mosquito blood meal, are activated to produce gametes. Gamete fusion in the insect midgut produces a zygote which develops into a motile ookinete, traversing the gut wall, and transforming into an oocyst, where 1000s of sporozoites are produced. The life cycle is closed when sporozoites, migrated from the ruptured oocyst to the mosquito salivary glands, are injected in a new human host by the insect bite.

The pathogenesis of malaria is caused by the asexual blood stages. In the clinical manifestations of *P. falciparum* malaria, the ability of parasites to sequester in the microvasculature of several organs, including the brain, is a major cause of disease severity, and of a fatal outcome (Miller et al., 2002; Milner et al., 2014; Sack et al., 2014). Consequently, the need to cure symptomatic patients traditionally drove efforts toward finding drugs targeting the asexual blood stage parasites, often underestimating the importance of eliminating also the sporozoite, and gametocyte transmission stages or, in *P. vivax*, the hypnozoites. The recent concerning reports from South East Asia of a decreased sensitivity of some *P. falciparum* infections to frontline combination therapies based on artemisinin derivatives is now calling for renewed efforts to address this emergency in the frame of a global strategy to control malaria and eventually eradicate this deadly parasite.

It is possible to cultivate all asexual and sexual blood stages of *P. falciparum in vitro*, unlike *P. vivax*. *Plasmodium* species infecting rodents have been also intensely studied as mouse models of aspects of malaria, with *P. berghei* particularly exploited for its amenability to genetic manipulation. In contrast, transgenesis technology has been comparatively more troublesome in *P. falciparum*. This review aims to highlight the importance of *Plasmodium* transgenic parasites, particularly those engineered with bioluminescent reporters, both in the study of the fundamental biology of *Plasmodium* and in developing effective antimalarial treatments.

Luciferase enzymes catalyze the light-producing chemical reactions of bioluminescent organisms, in which a luminogenic substrate (e.g., D-luciferin) is oxidized in the presence of ATP, yielding photons. These can be accurately measured by a luminometer with a sensitivity and a virtual absence of background that made bioluminescent reporters potent and versatile tools in biology (Smale, 2010). Luciferases hold a special place in the history of *Plasmodium* transgenesis: the first plasmid construct to be successfully transfected in malaria parasites contained a firefly (*Photynus pyralis*) luciferase gene whose expression, driven by the promoter of a parasite sexual stage-specific gene, was measured in ookinetes of the bird parasite *P. gallinaceum* (Goonewardene et al., 1993). Subsequently, luciferase reporters have been used to optimize transfection techniques in *Plasmodium* parasites (Epp et al., 2008; Hasenkamp et al., 2012), including the introduction of the luciferase from the sea pansy *Renilla reniformis*, where use of different substrates (D-luciferin and coelenterazine) enabled simultaneous detection of the two parasite produced reporters (Militello and Wirth, 2003; Helm et al., 2010). Since the 1990s, with the stable genetic transformation of different species of *Plasmodium* (Waters et al., 1997), luciferase reporter genes greatly contributed to elucidate key aspects of malaria infection, from the parasite cellular biology, protein trafficking, gene function, and drug resistance, in several developmental stages throughout the *Plasmodium* life cycle (**Figure 1**).

## The *Plasmodium* Life Cycle Marked by Bioluminescent Parasite Developmental Stages

### *Plasmodium* Mosquito Stages

Parasite sexual stage development in the mosquito vector is crucial for the transmission of *Plasmodium*, and elucidating the biology of this process may therefore lead to design novel malaria transmission-blocking strategies. Some studies with bioluminescent parasites highlighted the importance of post-transcriptional regulation acting on stability and translation of several mRNAs, including those encoding major proteins of the gamete and ookinete surface (Mair et al., 2006). Assays with luciferase reporters were for instance fundamental to identify regulatory elements in the transcripts of the P25 and P28 surface proteins of *P. gallinaceum* and *P. falciparum* (Golightly et al., 2000; Oguariri et al., 2006).

*Plasmodium* parasites expressing luciferases also improved tool development for applied studies. A powerful bioassay to determine parasite ability to infect mosquitoes is based on feeding cultured *Plasmodium* gametocytes to mosquitoes, and it is used to measure effect of transmission blocking drugs or antibodies. This assay is, however, technically demanding and time consuming as the resulting oocysts need to be individually counted in dissected insects. After improvements by using *P. berghei* parasites expressing a green fluorescent protein (GFP) in mosquito stages (Delves and Sinden, 2010), a transgenic line of the human parasite *P. falciparum* line expressing the firefly luciferase in oocysts was developed. In the resulting luminescence-based standard membrane feeding assay (SMFA) the mean luminescence intensity of individual and pooled mosquitoes accurately quantified mean oocyst intensity, eliminating the need for mosquito dissection, and putting the basis for significant SMFA scalability (Stone et al., 2014).

Toward the end of parasite development in the mosquito, the sporozoites produced in the oocyst migrate to the insect salivary glands. Number of salivary gland sporozoites, the only mosquito stages infectious to a mammalian host, is an important index of *Plasmodium* mosquito development. The construction of a *P. berghei* line where a GFP-luciferase fusion is specifically expressed in sporozoites enabled establishment of a simple and fast assay of sporozoite loads from whole mosquitoes (Ramakrishnan et al., 2012).

### Transmission from Mosquitoes: Sporozoites and Liver Stages

*Plasmodium* sporozoites injected from an infected mosquito to a human or rodent host start their intracellular development into the liver hepatocytes. This clinically silent stage is the target for prophylactic or vaccine strategies, particularly against *P. vivax* long lasting hypnozoites.

*Plasmodium* liver stage development has been poorly explored compared to that of blood stages partly because the *in vivo* and *in vitro* analyses, respectively, in mouse models and in cultured liver cells, are constrained by the necessity to sacrifice high numbers of mice or by inefficiency of sporozoite infection of cultured liver cells. Transgenic luciferase-expressing sporozoites improved detection strategies introducing bioluminescence imaging (BLI) and *in vivo* imaging system (IVIS) in the analysis of parasite liver stage development in live mice and in cultured hepatocytes. Real-time BLI requires injection of the luciferin substrate in the mouse or in the dissected organ and an intensified charge-coupled photon counting video camera to measure photon emission (Franke-Fayard et al., 2006; Braks et al., 2013). BLI and IVIS using firefly or sea pansy luciferases have been used for real-time, live monitoring of the progression of rodent parasitic infection in the whole animal or in specific organs (Ploemen et al., 2009; Annoura et al., 2013; Manzoni et al., 2014) and to test activity of drugs targeting liver stage infection, using *P. yoelii* and *P. berghei* transgenic sporozoites in human liver HepG2 or Huh-7 cells and in whole mice (Mwakingwe et al., 2009; Ramalhete et al., 2011, 2014; Derbyshire et al., 2012; Lacrue et al., 2013; Li et al., 2014; Marcsisin et al., 2014; Zuzarte-Luis et al., 2014). To improve these approaches, identification of parasite promoters specifically activated in liver development was achieved in *P. berghei*, also in this case relying on use of transgenic luciferase-promoter fusions (Helm et al., 2010).

The ability to reliably quantify parasite infection in hepatocytes is essential in the development of malaria vaccines. To overcome limitations of qRT-PCR-based quantification, *P. berghei* parasites expressing a GFP-luciferase fusion were introduced to evaluate antimalarial immunity both *in vivo,* in mice where this was induced by sporozoites unable to proliferate after irradiation or chloroquine prophylaxis, and *in vitro* in Huh-7 human liver hepatoma cells (Ploemen et al., 2011; Miller et al., 2013). Luciferase expressing *P. berghei* and *P. falciparum* sporozoites were also used to assess adequacy of sporozoite attenuation, obtained this time by genetic mutation, respectively, in *in vivo* murine malaria model and in primary human hepatocytes (Annoura et al., 2012; van Schaijk et al., 2014). These studies highlighted the role of cell mediated immunity mounting against the multiplication-deficient sporozoites. A role for antibody mediated immunity was instead shown by BLI of luciferase-expressing sporozoites of the human parasite *P. falciparum* in mice with a humanized liver, showing that infection in this organ was reduced by passive transfer of a monoclonal antibody targeting the sporozoite surface protein CSP (Sack et al., 2014). Finally, *P. berghei* and *P. yoelii* luciferase transgenic parasites were instrumental to evaluate modes of sporozoite administration, a critical bottleneck in immunization, and challenge protocols (Ploemen et al., 2013).

### From the Liver to the Blood: the Asexual Erythrocytic Stages

Maturation of the liver schizont releases 1000s of merozoites that invade blood stream erythrocytes and starts the asexual, symptomatic blood stage infection. In *P. falciparum* the blood stage schizonts disappear from circulation as they adhere to host ligands on endothelial cells of the microvasculature in several organs, especially in the brain and in the placenta, through parasite proteins expressed on the infected erythrocyte surface, leading to severe pathogenesis such as cerebral malaria or adverse effects during pregnancy. As parasites are observed to accumulate in several organs, including the brain, also in the mouse malaria model, real-time BLI in whole mice or in dissected organs were conducted with *P. berghei* transgenic lines expressing luciferase under a constitutive or a schizont-specific promoter to identify the involved components of the immune system (Franke-Fayard et al., 2005; Amante et al., 2007; Spaccapelo et al., 2010; Claser et al., 2011; Pasini et al., 2013; Imai et al., 2014).

The need to elucidate the mechanisms of malaria pathogenesis directed research on the fundamental biology of parasite asexual development, one important aspect being how the parasite regulates its gene expression. The extremely high A+T content of the *Plasmodium* genomes however, prevented homology based identification of promoters, regulatory elements, and parasite transcription factors, whereas luciferase reporters proved to be of paramount importance in functionally identifying gene promoters and regulatory regions (Horrocks and Kilbey, 1996; Porter, 2002; Militello et al., 2004; Hasenkamp et al., 2013a). This work identified sequences functioning as bi-directional promoters, like the intergenic region of the *P. berghei* elongation factor-1α (*ef-1α*) gene (de Koning-Ward et al., 1999; Fernandez-Becerra et al., 2003) or the intron of the *P. falciparum var* genes (Epp et al., 2008), or evaluated whether specific promoters from one *Plasmodium* species were able (Fernandez-Becerra et al., 2003; Ozwara et al., 2003), or unable (Azevedo and del Portillo, 2007) to recruit the transcriptional machinery of a different malaria species. Importantly, luciferase expressing parasites were used to identify regulatory regions governing the expression of the *P. falciparum* polymorphic *var* genes encoding the parasite sequestration ligands, whose expression switch is responsible for parasite antigenic variation, and immune evasion (Deitsch et al., 1999; Calderwood et al., 2003; Frank et al., 2006; Muhle et al., 2009). In summary, luciferase reporters not only contributed to identify functional elements involved in parasite gene regulation (Bischoff et al., 2000; Militello et al., 2004; López-Estraño et al., 2007; Gopalakrishnan and López-Estraño, 2010; Zhang et al., 2011; Patakottu et al., 2012), but also were essential to select specific promoters in the development of *Plasmodium* inducible expression systems (de Azevedo et al., 2012; Kolevzon et al., 2014) and to test new regulatory regions in chromosomally integrated luciferase cassettes (Ekland et al., 2011; Weiwer et al., 2011; Che et al., 2012; Khan et al., 2012; Hasenkamp et al., 2013b).

A major effort in the fight against malaria, particularly *P. falciparum*, has been the screening for new antimalarial drugs, an endeavor that the appearance of artemisinin resistance in South East Asia makes dramatically urgent. In the past decades, *in vitro* methods measuring the incorporation of [^3^H]-labeled hypoxanthine and ethanolamine or the activity of parasite Lactate Dehydrogenase have been the standard for *P. falciparum* cell based assays and used in large drug screenings (Fidock, 2010). The demand for high-throughput, non-radioactive assays prompted to exploit also in *Plasmodium* the high sensitivity and virtual absence of background of luciferase reporters, until recently used in this field only to study expression of the *P. falciparum* multidrug resistance gene *pfmdr1* in drug treated parasites (Myrick et al., 2003; Waller et al., 2003). To this aim a *P. falciparum* line expressing the firefly luciferase under the *heat shock protein 86* (*pfhsp86*) gene promoter in asexual stages enabled establishment of a cell-based luciferase drug screening assay in 96w plates (Cui et al., 2008), subsequently adapted to 384w plate using 10^5^–10^6^ parasites per well (Lucumi et al., 2010). Also *P. berghei* parasites expressing a firefly luciferase-GFP fusion were used for *in vitro* and *in vivo* bioluminescence drug assay, enabling use of animal models to test new drugs *in vivo* (Franke-Fayard et al., 2008; Lin et al., 2013).

### Preparing Departure from the Blood: the Gametocytes

*Plasmodium* gametocytes are the parasite sexual stages responsible for the transmission from the vertebrate host to the mosquito. Male and female gametocytes are formed in the bloodstream and, in *P. falciparum*, they mature in 10 days through five developmental stages. Upon ingestion in the mosquito gut, mature gametocytes promptly differentiate into gametes and fertilization ensures parasite infection in the insect vector. A key priority in the present goal to globally eliminate malaria is to identify new drugs targeting in the bloodstream both the asexual and the sexual stages of the parasite. However, the non-replicative nature of gametocytes imposed to develop specific cell based screening assays, different from those used for asexual stages. One problem is for instance that of false negative signals due to the persistence of fluorescent reporter or of parasite enzyme activities in unhealthy or dying gametocytes. *P. falciparum* lines expressing a GFP-firefly luciferase gene under gametocyte specific promoters were established (Adjalley et al., 2011) and used in high-throughput screening assays of compounds with anti-asexual stage activity (Lucantoni et al., 2013). Recently, luciferase-based gametocyte assays have been improved by replacing the commonly used commercial luciferase substrates with an ATP-free, non-lysing D-luciferin formulation, yielding assay readouts that more reliably monitored viability and sensitivity to compounds of the treated gametocytes (Cevenini et al., 2014). In this work, absence of parasite cell lysis and the introduction in *P. falciparum* of the use of a potent luciferase from *Pyrophorus plagiophthalamus* under a gametocyte promoter enabled to perform for the first time BLI at the level of single parasite cells, individually distinguishing live and dead *P. falciparum* gametocytes (Cevenini et al., 2014).

## Multiplexing, Subcellular Localization, Imaging: the Future in the Use of Bioluminescent Malaria Parasites

Virtually all stages of the complex life cycle of malaria parasites have been enlightened by the use in several studies of luciferase reporters. These engineered parasites provided key answers to fundamental biological questions and now represent important tools for drug screening. Novel potent reporters have already expanded the luciferase repertoire used in *P. falciparum* beyond the *P. pyralis* and Renilla enzymes (Azevedo et al., 2014; Cevenini et al., 2014), increasing sensitivity and enabling to further reduce parasite numbers in high-throughput screening assays, a non-trivial improvement when using specific stages (e.g., the gametocytes) whose cultivation is technically demanding. Nevertheless exploitation of the full potential of bioluminescent reporters in malaria research is just moving the first steps.

The possibility to tune luciferase emission properties, such as emission wavelength, kinetics or termo- and pH-stability, via random or site-directed mutagenesis or use of enzyme natural variants, led to introduce multicolor bioluminescence in antimalarial drug screening. A green and a red light emitting luciferase from *P. plagiophthalamus* were expressed in *P. falciparum* immature and mature gametocytes, providing for the first time the possibility to simultaneously measure differential, stage specific effects of drugs in a dual-color luciferase assay, and opening the possibility to apply multicolor bioluminescence to any parasite stage in fundamental and applied studies. A dual expression system with distinct luciferases would for instance be valuable in cell based high-throughput screenings to readily identify and discard compounds active against the reporter rather than the target cell (Thorne et al., 2012), as they will most likely affect only one luciferase type.

Another promising application of luciferase reporters is through their fusion to specific signals used by the parasite to traffic proteins in different extra cellular compartments of the infected erythrocyte. As protein export is uniquely regulated in the parasite and is essential for its survival, use of such fusions may be invaluable to screen for compounds targeting this process. Preliminary studies were conducted with the *P. pyralis* enzyme (Burghaus and Lingelbach, 2001) and more recent work established *P. falciparum* lines which express a brighter deep-sea shrimp luciferase equipped with sequences driving the reporter in the parasite cytoplasm or in erythrocyte compartments (Azevedo et al., 2014).

In another field of application, the achievement of single parasite cell BLI and the availability of luciferases whose red-shifted light emission is more efficiently detectable from blood and tissues are paving the road to significant progress in analyses of the host-parasite interplay. Co-cultures of different *P. falciparum* stages and human cell types *in vitro* can provide new insights of the physiology of asexual and sexual stage parasite sequestration. The increased sensitivity achieved in *in vivo* mouse imaging with a red-shifted luciferase expressed by the unicellular protozoan parasite *Trypanosoma brucei* (Van Reet et al., 2014) is promising in view of use also in *Plasmodium* infected mice. Importantly, the increasing availability of humanized mouse models for *P. falciparum* and *P. vivax* infections, supporting development of asexual, and sexual blood stages and of liver stages (Kaushansky et al., 2014) and the use of *P. falciparum* transgenic lines with a luciferase expressed constitutively (Vaughan et al., 2012) or under stage-specific promoters are expected to answer many unsolved questions.

The wealth of biological information provided by the use of engineered bioluminescent malaria parasites, not to mention those not reviewed here expressing a variety of fluorescent reporters, has been and will most likely continue to be enormous. The confined use of these whole cell biosensors in laboratory settings does not pose regulatory concerns on environmental release. From their aseptic sites of utilization, these genetically modified parasites will nevertheless have the most significant impact in the real world, contrasting the unbearable burden of a worldwide devastating disease.

## Author Contribution

GS drafted and PA edited the manuscript.

## Conflict of Interest Statement

The authors declare that the research was conducted in the absence of any commercial or financial relationships that could be construed as a potential conflict of interest.
